# Use of 27G needles improves sensitivity and performance of ATCC anaerobe reference microorganism detection in BacT/Alert system

**DOI:** 10.1016/j.omtm.2021.01.010

**Published:** 2021-01-26

**Authors:** Salvatore Pasqua, Giampiero Vitale, Anna Pasquariello, Bruno Douradinha, Fabio Tuzzolino, Francesca Cardinale, Chiara Cusimano, Chiara Di Bartolo, Pier Giulio Conaldi, Danilo D’Apolito

**Affiliations:** 1 Unità Prodotti Cellulari (GMP), Fondazione Ri.MED c/o IRCCS-ISMETT (Istituto Mediterraneo per i Trapianti e Terapie ad Alta Specializzazione), Via E. Tricomi 5, 90127 Palermo, Italy; 2 Unità di Medicina di Laboratorio e Biotecnologie Avanzate, IRCCS-ISMETT, Via E. Tricomi 5, 90127 Palermo, Italy; 3 Unità Medicina Rigenerativa ed Immunologia, Fondazione Ri.MED c/o IRCCS-ISMETT, Via E. Tricomi 5, 90127 Palermo, Italy; 4 Ufficio Ricerca, IRCCS-ISMETT, Via E. Tricomi 5, 90127 Palermo, Italy

**Keywords:** microbiological contaminations, automated blood culture system, BacT/Alert, sterility testing, microorganisms, hospital infections, method validation, quality control, good manufacturing practice, European Pharmacopoeia

## Abstract

Effective detection of microbiological contaminations present in medicinal cellular products is a crucial step to ensure patients’ safety. In recent decades, several rapid microbiological methods have been developed and validated, but variabilities linked to the use of different resources have led to discordant validation of methods and performance results. Considering this, while developing an in-house BacT/Alert-based method, we evaluated all of the materials used in its validation. Of particular importance, we noticed that the syringe gauge used to inject the samples into the bottles was crucial to obtain robust results. We chose to conduct a comparative test between the BacT/Alert system and the compendial method described in the European Pharmacopoeia, using five dilutions of nine reference microorganism strains and 21G or 27G needles. Our results confirmed that the BacT/Alert system is a valid and faster alternative method to assess sterility of clinical cell therapy products, and that the use of 27G needles increases its sensitivity to detect reference anaerobe microorganisms.

## Graphical Abstract

**Figure:**
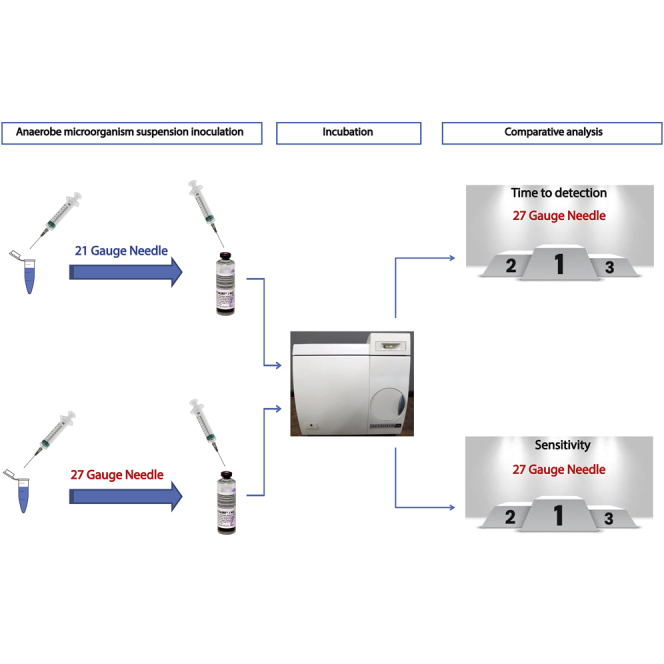

## Teaser

The BacT/Alert system is used in GMP control activities to test the sterility of ATMPs. The use of 27G needles decreases the TTD of most of the tested microorganisms, when compared to the European Pharmacopeia method, and allows less air intrusion and improves the growth and sensitivity of the method for anaerobic microorganisms.

## Introduction

Pharmaceutical drugs must undergo several quality checks that ensure sterility, safety, efficacy, and identity before becoming available on the market. These controls are regulated by international pharmacopoeias. These regulations were initially created to control the production of classical drugs by pharmaceutical companies, which are produced in large batches and do not have particular time restrictions before their commercialization. However, the same approach cannot be applied to most advanced therapy medicinal products (ATMPs),^1^ which, due to their characteristics, are produced in batches of more limited quantities, have a shorter shelf life, and require faster quality controls (QCs) than traditional drugs. Due to their short shelf life, ATMPs are administered to patients immediately after passing the QC in-process control tests. Of particular importance, ATMPs are very heterogeneous, and thus the QC strategy is mostly defined on a case-by-case basis. To fully guarantee the patient’s safety, QC release tests are done after the formulation of ATMPs. However, the results of such tests may take several days to be known.^1^^,^^2^ Development and optimization of in-house QC analytical methods in health facilities benefit from synergistic collaborative efforts with microbiological diagnostics laboratories, especially regarding the sterility of ATMPs.^3^ The alternative to in-house procedures is to send the samples to be analyzed by an external good manufacturing practices (GMPs)-accredited laboratory; however, this option is time-consuming and leads to incremental costs.^4^ Validation of an alternative microbiological method is done according to international regulations, which state that a potential alternative method must be performed in parallel to the respective golden standard method. Analysis of the results obtained will show if the performance of the alternative method is the same or better than the one shown by the classical method.^5^, ^6^, ^7^, ^8^, ^9^ Several alternative rapid microbiological methods have been developed, and many clinical laboratories have validated alternative procedures based on blood culture systems already present in laboratories, such as Bactec and BacT/Alert.^3^^,^^4^^,^^10^, ^11^, ^12^, ^13^, ^14^, ^15^, ^16^, ^17^ However, reported validations of alternative analytical methods using Bactec and BacT/Alert do not follow a standardized protocol,^10^, ^11^, ^12^, ^13^, ^14^ i.e., each laboratory used different microorganisms, titers, growth media, and other variables according to their specific needs or experience. Thus, despite their potential better performance, these automated systems can give different inter-laboratory results, due to the lack of a standardized protocol. This means that each laboratory should perform validations by analyzing in detail all of the possible factors that can affect the method. To the best of our knowledge, no systematic study has considered variabilities linked to the use of different reagents or materials during the validation phases.

Although most of the microorganisms isolated in cleanrooms are aerobic or facultative aerobic, it has been shown that anaerobic microorganisms can also be found in this environment, tolerating non-ideal growth conditions for them.^18^ Also, biological material used in the production of ATMPs, such as cord or peripheral blood, might be contaminated with anaerobic microorganisms, resulting in adulteration of the final product.^12^^,^^19^ Thus, anaerobic microorganisms must be also considered when assessing the sterility of potential ATMPs.^18^^,^^20^

Our study compares the direct inoculation method^21^^,^^22^ and the alternative BacT/Alert-based method by performing a systematic study taking into account variabilities induced to the use of the different materials.^5^^,^^8^^,^^23^^,^^24^ Our findings confirmed that the BacT/Alert-based method could be used as an alternative sterility test method. Furthermore, we have shown that the use of a gauge of smaller diameter improves the performance of the BacT/Alert system for the anaerobic microbial species tested. These results suggest that 27G needles could be implemented in routine activities of pharmaceutical sterility tests to improve the detection of anaerobic microorganisms in potential ATMPs.

## Results

### Validation

#### Specificity

In order to demonstrate the specificity of the alternative BacT/Alert method, we performed a growth promotion test (GPT) inoculating 25–50 colony-forming units (CFU) of the microorganisms detailed in Table 1.^5^^,^^21^ The results are summarized in Tables 2 and 3. For each medium, we determined its ability to promote the growth of each microorganism. We did not find any difference between tryptic soy broth (TSB) and BacT/Alert *iAST* media in terms of promotion of the growth of aerobic bacteria and fungi (Table 2).

**Table 1:** Microbial strains used in this work

Microorganisms tested in aerobic media	Microorganisms tested in anaerobic media
*S. aureus* ATCC 6538	*C. sporogenes* ATCC 19404
*P. aeruginosa* ATCC 9027	*B. fragilis* ATCC 25285
*B. subtilis* ATCC 6633	*S. pyogenes* ATCC 19615
*S. epidermidis* ATCC 12228	
*S. pyogenes* ATCC 19615	
*C. albicans* ATCC 10231	
*A. brasiliensis* ATCC 16404

**Table 2:** Recovery and TTD for microorganisms in aerobic media

Microorganism	Gauge	Range of total CFU inoculated									
		25–50		5–10		2–5		1–2		0–1	
		Recovery	TTD (h) ± SD	Recovery	TTD (h) ± SD	Recovery	TTD (h) ± SD	Recovery	TTD (h) ± SD	Recovery	TTD (h) ± SD
*S. aureus* (*iAST*)	21	12/12	32.2 ± 3.6	12/12	38.6 ± 4.7	9/12	41.3 ± 6.5	6/12	38.8 ± 5.3	2/12	40.8 ± 3.4
	27	12/12	31.8 ± 6.8	12/12	33.4 ± 3.6	12/12	36 ± 4.9	8/12	40.5 ± 6.1	3/12	39.2 ± 7.3
*S. aureus* (TSB*)*	21	12/12	32 ± 11.8	12/12	28 ± 9.3	10/12	28.8 ± 10.1	10/12	33.6 ± 12.4	5/12	28.8 ± 10.7
	27	12/12	32 ± 11.8	12/12	30 ± 10.8	12/12	26 ± 6.9	6/12	32 ± 12.4	3/12	32 ± 13.8
*P. aeruginosa* (*iAST*)	21	12/12	25.4 ± 2.4	12/12	60.2 ± 12.2	9/12	64 ± 12	6/12	83.2 ± 12.4	0/12	none
	27	12/12	24.2 ± 2.4	12/12	53.8 ± 21.3	12/12	66.7 ± 8.5	6/12	70.4 ± 21.2	0/12	none
*P. aeruginosa* (TSB)	21	12/12	48 ± 0	12/12	62 ± 12.3	6/12	68 ± 9.8	4/12	72 ± 0	0/12	none
	27	12/12	60 ± 12.5	12/12	64 ± 11.8	7/12	61.7 ± 12.8	5/12	62.4 ± 13.1	1/12	72 ± 0
*B. subtilis* (*iAST*)	21	12/12	12 ± 0	12/12	13.8 ± 1.1	12/12	14.2 ± 0.7	10/12	15.12 ± 1.2	5/12	14.4 ± 0
	27	12/12	12.2 ± 0.7	12/12	15 ± 2.7	12/12	15.4 ± 3.5	10/12	15.12 ± 1.2	5/12	15.4 ± 1.3
*B. subtilis* (TSB)	21	12/12	48 ± 0	12/12	48 ± 0	11/12	48 ± 0	9/12	48 ± 0	2/12	48
	27	12/12	54 ± 10.85	12/12	58 ± 21.6	11/12	69.8 ± 19.9	8/12	75 ± 29.91	2/12	96 ± 33.9
*S. epidermidis* (*iAST*)	21	12/12	30.2 ± 2.2	12/12	35 ± 6.4	12/12	33.8 ± 5.2	1/12	40.8 ± 0	1/12	48 ± 0
	27	12/12	29.6 ± 2.4	12/12	35.4 ± 9.5	12/12	34.6 ± 7.3	2/12	34.8 ± 1.7	3/12	44 ± 6
*S. epidermidis* (TSB)	21	12/12	144 ± 14.5	12/12	160 ± 18.7	9/12	186.6 ± 10.6	0/12	none	0/12	none
	27	12/12	144 ± 20.5	12/12	162 ± 23.2	9/12	186.6 ± 10.6	1/12	168 ± 0	0/12	none
*S. pyogenes* (*iAST*)	21	12/12	36.2 ± 8.7	12/12	38.4 ± 8.4	7/12	61 ± 12	4/12	80.4 ± 23.2	0/12	none
	27	12/12	23 ± 1.2	12/12	25 ± 2.2	9/12	26.1 ± 2.8	6/12	26.8 ± 2.4	2/12	26.4 ± 0
*S. pyogenes* (TSB)	21	12/12	48 ± 0	12/12	48 ± 0	12/12	52 ± 9.3	6/12	60 ± 13.1	0/12	none
	27	12/12	48 ± 0	12/12	64 ± 21.3	11/12	65.4 ± 18.9	11/12	69.8 ± 16.8	1/12	72 ± 0
*C. albicans* (*iAST*)	21	12/12	39.8 ± 4.4	12/12	44.4 ± 3.8	12/12	77.4 ± 12.2	12/12	71 ± 12.1	0/12	none
	27	12/12	38.8 ± 5.7	12/12	43.6 ± 3.8	12/12	63.2 ± 10.2	12/12	63.2 ± 10.2	2/12	81.6 ± 3.4
*C. albicans* (TSB)	21	12/12	90 ± 10.8	12/12	86 ± 16.1	12/12	96 ± 0	12/12	96 ± 0	0/12	none
	27	12/12	84 ± 21.7	12/12	90 ± 10.8	12/12	84 ± 12.5	12/12	94 ± 6.9	0/12	none
*A. brasiliensis* (*iAST*)	21	12/12	62.8 ± 5.3	12/12	65.4 ± 3.8	12/12	71.2 ± 8.4	8/12	83.4 ± 8	0/12	none
	27	12/12	57.8 ± 8.9	12/12	67.6 ± 7.6	12/12	68.4 ± 6.5	8/12	81.3 ± 9.3	0/12	none
*A. brasiliensis* (TSB)	21	12/12	90 ± 10.85	12/12	86 ± 16.04	12/12	96 ± 0	12/12	104 ± 11.8	0/12	none
	27	12/12	76 ± 13.8	12/12	74 ± 6.9	12/12	80 ± 11.8	10/12	100.8 ± 18.9	0/12	none

TTD, time to detection; SD, standard deviation.

**Table 3:** Recovery and TTD for microorganisms in anaerobic media

Microorganism	Gauge	Range of total CFU inoculated									
		25–50		5–10		2–5		1–2		0–1	
		Recovery	TTD (h) ± SD	Recovery	TTD (h) ± SD	Recovery	TTD (h) ± SD	Recovery	TTD (h) ± SD	Recovery	TTD (h) ± SD
*B. fragilis* (*iNST*)	21	5/12	95.5 ± 32.7	3/12	116.8 ± 65.8	3/12	78.4 ± 20	0/12	none	0/12	none
	27	12/12	61.6 ± 17.1	12/12	72 ± 13.5	8/12	77.1 ± 1.5	5/12	78.2 ± 2.1	0/12	none
*B. fragilis* (FTM)	21	12/12	60 ± 12.5	12/12	70 ± 6.9	10/12	74.4 ± 13.6	5/12	76.8 ± 10.7	4/12	78 ± 12
	27	12/12	58 ± 16.1	8/12	63 ± 17.8	7/12	61.7 ± 12.8	4/12	66 ± 12	0/12	none
*C. sporogenes* (*iNST*)	21	12/12	32.2 ± 10	12/12	36 ± 10.7	11/12	47.8 ± 16	5/12	76.32 ± 20.7	0/12	none
	27	12/12	25 ± 4.1	12/12	26.4 ± 4.1	12/12	27.4 ± 4.4	12/12	29.4 ± 2.9	2/12	25.2 ± 5.1
*C. sporogenes* (FTM)	21	12/12	24 ± 0	12/12	24 ± 0	12/12	24 ± 0	12/12	26 ± 6.92	0/12	none
	27	12/12	24 ± 0	12/12	24 ± 0	12/12	24 ± 0	12/12	24 ± 0	4/12	24 ± 0
*S. pyogenes* (*iNST*)	21	12/12	35.6 ± 9.4	12/12	37 ± 10	7/12	59.3 ± 13	5/12	76.3 ± 20.7	0/12	none
	27	12/12	17.8 ± 1.2	12/12	18.8 ± 0.9	12/12	20.7 ± 1.2	8/12	23.7 ± 4.7	3/12	4 ± 1.4
*S. pyogenes* (FTM)	21	12/12	48 ± 0	12/12	48 ± 0	12/12	48 ± 0	6/12	60 ± 13.1	0/12	none
	27	12/12	48 ± 0	12/12	48 ± 0	12/12	48 ± 0	7/12	51.42 ± 9.1	1/12	72 ± 0

For anaerobic bacteria, we observed that *Clostridium sporogenes* and *Streptococcus pyogenes* grew similarly in both fluid thioglycollate medium (FTM) and *iNST* media, for both 21G and 27G needles (Table 3). Alternatively, as described above, *Bacteroides fragilis* grew differently in these media. In particular for FTM, we did not observe any difference for both gauges. Using the *iNST* medium, inoculation of *B. fragilis* with a 21G needle led to poor growth (5 bottles positive out of 12), while growth was observed in all bottles when bacteria were administered with a 27G needle (Table 3). A preliminary GPT done with *Propionibacterium acnes* showed complete recovery of this slow-growing anaerobic bacterium either in FTM or *iNST* media (Table S1).

#### Detection limit

Tables 2 and 3 resume the results obtained regarding detection limit determination. Using both TSB and *iAST* media, we observed similar detection limits for aerobic bacteria and fungi using both gauges (Tables 2 and S2). *Staphylococcus aureus*, *Bacillus subtilis*, *Candida albicans*, and *Aspergillus brasiliensis* showed a detection limit of 1–2 CFU. *Pseudomonas aeruginosa* showed a detection limit of 1–2 in *iAST* and of 2–5 CFU in TSB. For *Staphylococcus epidermidis*, a detection limit of 2–5 CFU was observed in both media. *S. pyogenes* had a detection limit of 1–2 CFU in TSB. The only microorganism that displayed different detection limits regarding the gauge used was *S. pyogenes*, which, when grown in *iAST*, showed detection limits of 2–5 CFU and 1–2 CFU for 21G and 27G needles, respectively.

Different media for anaerobic bacteria showed diverse detection limits for the three anaerobes tested (Tables 3 and S3). For FTM, we did not observe any differences regarding growth when anaerobic microorganisms were inoculated with either a 21G or 27G needle. When this medium was used, *B. fragilis* showed a detection limit of 2–5 CFU, while for *C. sporogenes* and *S. pyogenes* it was 1–2 CFU, for both gauges used. For *iNST*, the use of a 27G needles was fundamental for the recovery of anaerobic microorganisms and to improve the detection limit. The detection limit for *C. sporogenes* and *S. pyogenes* was 1–2 CFU using a 27G needle, and 2–5 CFU when a 21G needle was used. For *B. fragilis*, the detection limit was 2–5 CFU and was only achieved with a 27G needle. When *B. fragilis* was inoculated using a 21G needle, it did not reach 50% recovery, even when 25–50 CFU were used (Tables 3 and S3).

The number of a microorganism’s CFU was confirmed by plating 100 μL of each dilution in appropriate solid media plates and was in agreement with the range of CFU used in this validation (data not shown).

#### Technical cross-contaminations

To evaluate potential cross-contaminations, for each experiment and operator, we handled 15 positive samples and 5 negative samples (peptone water). The results showed no growth in the negative samples. This result demonstrated that all phases of the process were performed in sterility conditions and did not lead to false positives in the negative samples.

#### Ruggedness and repeatability

To evaluate the ruggedness and the repeatability of the alternative BacT/Alert method, we took into consideration the results obtained from GPTs using BacT/Alert, where *iAST* and *iNST* media were inoculated with 25–50 CFU of the tested microbial species. Tables 2 and 3 summarize the results obtained from four different operators working in 4 different days and using different media lots. Since all microorganisms grew in all inoculated bottles (except for *B. fragilis* inoculated with a 21G needle), the BacT/Alert-based method produced robust and reproducible results.

#### Time to detection (TTD)

To analyze the TTD, we considered the values of the inoculations using 25–50 CFU. We chose this range of inoculation because it allowed the growth of all cultured microorganisms and displayed less variability. As detailed in Table 4, all aerobic microorganisms grew faster in *iAST* than in TSB, with the exception of *S. aureus*, which grew similarly. Different gauges did not affect TTD of most of the inoculated aerobic microorganisms. However, some microorganisms showed a significant lower TTD when diverse gauges were used, such as *P. aeruginosa* inoculated with a 21G needle and *A. brasiliensis* with a 27G needle, both grown in TSB (p = 0.0068 and p = 0.0114, respectively), and *S. pyogenes* grown in *iAST* inoculated with a 27G needle (p = 0.0003).

**Table 4:** Statistical analysis of TTD for microorganisms in aerobic media

Microorganism	Gauge	Range of total CFU inoculated: 25–50		
		TTD (h) ± SD		
		*iAST*	TSB	p
*S. aureus*	21	32.2 ± 3.6	32 ± 11.8	0.9561
	27	31.8 ± 6.8	32 ± 11.8	0.9599
	P	0.8592	1.0000	
*P. aeruginosa*	21	25.4 ± 2.4	48 ± 0	0.0000
	27	24.2 ± 2.4	60 ± 12.5	0.0000
	P	0.2336	0.0068	
*B. subtilis*	21	12 ± 0	48 ± 0	0.0000
	27	12.2 ± 0.7	54 ± 10.85	0.0000
	P	0.3436	0.0818	
*S. epidermidis*	21	30.2 ± 2.2	144 ± 14.5	0.0000
	27	29.6 ± 2.4	144 ± 20.5	0.0000
	P	0.5298	1.0000	
*S. pyogenes*	21	36.2 ± 8.7	48 ± 0	0.0007
	27	23 ± 1.2	48 ± 0	0.0000
	P	0.0003	1.0000	
*C. albicans*	21	39.8 ± 4.4	90 ± 10.8	0.0000
	27	38.8 ± 5.7	84 ± 21.7	0.0000
	P	0.6352	0.4037	
*A. brasiliensis*	21	62.8 ± 5.3	90 ± 10.85	0.0000
	27	57.8 ± 8.9	76 ± 13.8	0.0009
	P	0.1087	0.0114	

Regarding anaerobic microorganisms (Table 5), TTD varied according with the gauge used for inoculation. *B. fragilis* and *C. sporogenes* inoculated using 21G needles showed lower TTD in FTM than in *iNST* (p = 0.0034 and p = 0.0161, respectively), while *S. pyogenes* displayed higher TTD in the same conditions (p = 0.0008). Also, *S. pyogenes* was the only strain that showed a lower TTD in *iNST* when inoculated via a 27G needle (p < 0.0001). Interestingly, *B. fragilis* and *C. sporogenes* inoculated by 27G needles grew faster in *iNST* when compared to inoculations performed with 21G needles (p = 0.0056 and p = 0.0155, respectively). In agreement, preliminary tests done with *P. acnes* showed lower TTD using *iNST* medium (Table S1). Also, the use of 27G needles led to a significant lower TTD (p = 0.0014).

**Table 5:** Statistical analysis of TTD for microorganisms in anaerobic media

Microorganism	Gauge	Range of total CFU inoculated: 25–50		
		TTD (h) ± SD		
		*iNST*	FTM	p
*B. fragilis*	21	95.5 ± 32.7	60 ± 12.5	0.0034
	27	61.6 ± 17.1	58 ± 16.1	0.6008
	P	0.0056	0.7372	
*C. sporogenes*	21	32.2 ± 10	24 ± 0	0.0161
	27	25.6 ± 4.8	24 ± 0	0.2728
	P	0.0155	1.0000	
*S. pyogenes*	21	35.6 ± 9.4	48 ± 0	0.0008
	27	17.8 ± 1.2	48 ± 0	0.0000
	p	0.0000	1.0000	

#### Analytical method equivalency

Regarding detection limit, both methods were equivalent for all microorganisms when using 27G needles (Tables S2 and S3), except for *P. aeruginosa*, to which the alternative method showed a better detection limit. Considering the use of a 21G needle, both methods were considered equivalent when growing aerobic microorganisms, except for *P. aeruginosa*, which had a better detection limit when grown in BacT/Alert *iAST*, and *S. pyogenes*, with a better detection limit when the compendial method was used.

Concerning the TTD, for the aerobic microorganisms and both gauges, the BacT/Alert method showed better results when compared to the European Pharmacopeia (Eur Ph) method, except for *S. aureus*, which was equivalent (Table 4). As stated above, performance of both methods for TTD evaluation of anaerobic microorganisms was influenced by the type of gauge used. With a 21G needle, the compendial method showed better TTD for *B. fragilis* and *C. sporogenes*. Per turn, a 27G needle restored the equivalence of TTD for both methods using *B. fragilis* and *C. sporogenes* (Table 5). The TTD of *S. pyogenes* was better with a 27G needle and using the alternative method (Table 5). *P. acnes* showed also a better TTD when the alternative method was used, for both 21G and 27G needles (Table S1).

## Discussion

International pharmacopeias regulate standard analytical methods for quality control of ATMPs. Alternative methods are accepted when the results of their methodological validation show that their performance is comparable to or better than the golden standard method.^7^, ^8^, ^9^^,^^25^ Recently, it has been shown that, in addition to having a performance comparable to the golden standard method, automatic systems for blood cultures are able to decrease the detection time (TTD) of the microorganisms tested.^13^^,^^14^ To perform comparability studies, pharmacopoeias require validation studies to be done using well-defined microorganisms strains.^5^^,^^6^^,^^22^ However, these microbial reference strains may not represent all of the microorganisms that operators or materials could introduce into production environments or that may be present in the original biological sample from which ATMPs are produced. For this reason, during validation, it is advisable to gradually extend it to potential contaminants, such as environmental bacteria and fungi or microbial contaminants of previously used cellular products. This allows a more comprehensive evaluation of the performance of the alternative method, as recommended in Eur Ph 2.6.27.^5^ To date, few laboratories have published validation data using broad sets of microorganisms. Although it has been demonstrated that automatic systems can effectively detect contamination of biological samples by both aerobic and anaerobic microorganisms, these studies have been done by different laboratories using diverse protocols.^10^, ^11^, ^12^, ^13^, ^14^ The use of different reagents or materials may lead to non-reproducible protocols when comparing inter-laboratory procedures, which could be avoided by defining an international standardized protocol, as done for detection of mycoplasma contamination.^2^^,^^26^^,^^27^ Our scope was to compare the direct inoculation method^21^^,^^22^ and a BacT/Alert-based method^5^^,^^6^^,^^8^ by performing a systematic study taking into account the variability linked to the use of the different reagents or materials. We performed several preliminary experimental tests to identify potential critical points (Tables S4–S10). First, we confirmed that all liquid and solid media necessary for this validation supported the growth of the selected microorganisms, as required by the Eur Ph.^5^^,^^21^ As required by GMP guidelines,^28^ the viability, identity, and titer of all reference microorganisms (Table 1) were confirmed before the validation and were in agreement with those provided by the certificate of analysis (CoA), showing no major variabilities (data not shown). We and others have confirmed the microorganism information given by the CoA provided by the supplier, confirming the suitability of their products for microbiological validation strategies.^29^^,^^30^

We also evaluated which suspension medium would be suitable for our validation protocol. Cellular therapy products are grown in complex media, which contain many components that can interfere with microbial growth (e.g., antibiotics).^1^ However, previous studies have shown that microorganism growth detection is not affected by the suspension medium used.^10^^,^^12^ In agreement, we compared the growth of microorganisms in peptone water and in exhausted culture medium and found no significant differences (Table S10). Thus, we chose peptone water for the validation of the alternative BacT/Alert method.

We found that the shape of the container in which the suspensions were prepared, and the gauge used to inoculate the sample inside the bottles were of particular importance. The gauge of the needle affected the growth of anaerobic bacteria inside the blood culture bottles, possibly through an undesired introduction of air due to the diameter of the needle.^24^ In fact, using gauges of different diameters (21G, 23G, 25G, and 27G), we noticed an improvement on the detection of anaerobic microorganisms and, consequently, in the experimental repeatability as the gauge increased, i.e., decreasing the needle diameter (Table S9). For the validation procedure we focused on the 21G and 27G needles to understand how the needle diameter could affect the method’s performance. When the nine reference microorganisms and *S. epidermidis* (which, due to its presence in human skin,^20^ may contaminate clean room surfaces) were inoculated at low levels (25–50 CFU)^5^^,^^21^^,^^22^ using a 27G needle, we observed a full recovery, i.e., all microorganisms grew in all inoculated bottles, for both Eur Ph and BacT/Alert methods (Tables 2 and 3). Similar results were obtained using a 21G needle (Tables 2 and 3), except for *B. fragilis* in *iNST* (Table 3). Since *B. fragilis* grew in all bottles containing *iNST* media when inoculated with a 27G needle, we assume that the poor outcome in recovery in the same conditions but using a 21G needle, which has a larger diameter, led to unwanted intrusion of air, which affected the growth of this bacterium, as suggested elsewhere.^24^

To validate our strategy, we assessed its specificity, detection limit, ruggedness, repeatability, and cross-reaction contaminations and compared them with those obtained with the Eur Ph classical method. Specificity was confirmed by the ability of all tested microorganisms to grow in all selected media. No variable results were observed even when the two methods were performed by four different operators in 4 diverse days and using different media lots, confirming the ruggedness and repeatability of both Eur Ph and BacT/Alert alternative methods. Simultaneous incubation of contaminated and negative samples showed no cross-reaction contamination, since no false negatives or false positives were found, as expected. Regarding detection limit, the two methods showed similar results for the aerobic microorganisms used when inoculated with both 21G and 27G needles (Tables 2 and S2) and for the anaerobic microorganisms using 21G needles (Tables 3 and S3). However, a lower detection limit was observed for the anaerobic microorganisms, including *P. acnes*, and for *S. pyogenes*, a facultative anaerobic bacterium (Tables 2, 3, S1, S2, and S3), when compared to the results obtained with the Eur Ph method. Overall, the BacT/Alert method using 27G needles satisfies all requirements stated by the Eur Ph for an alternative method regarding specificity, ruggedness, detection limit, repeatability, and cross-reaction contaminations.

Detailed analysis of the TTDs of each microorganism showed that the aerobic microorganisms grew faster in *iAST* than in TSB, with the exception of *S. aureus*, which grew similarly (Table 4). For anaerobic microorganisms, only the use of a 27G needle allowed us to obtain comparable results between the two methods. In particular, when using this gauge we observed a faster growth in *iNST* (Tables 5 and S1). We assume that this result is due to the different characteristics of the membrane rubber that constitutes the inoculation septum. The membrane of a FTM bottle is thicker than that of a *iNST* bottle, which does not allow an unwanted entry of air in the former, regardless of the gauge used. As mentioned above, the potential higher input of air into the BacT/Alert bottles inoculated with the 21G needle negatively affects the growth of anaerobic microorganisms.^24^ In fact, *S. pyogenes*, which is a facultative anaerobe, is less affected by this possible air intrusion, but also grows faster when inoculated with a 27G needle (Tables 4 and 5). As expected, different microorganisms have different TTDs and frequency recovery values, strengthening the importance to test other microorganisms that may contaminate either cleanroom surfaces or the pharmaceutical product, as suggested in Eur Ph 2.6.27.^5^

The temperature range recommended by Eur Ph for the alternative methods is 35°C–37°C.^5^ Accordingly, the BacT/Alert method was done at 36°C. Interestingly, another study compared the effect of two different temperatures (25°C and 35°C) on the growth of many microorganisms using BacT/Alert.^14^ This approach increases the specificity of the method due to a heterogeneous growth rate of microorganisms at different temperatures. It would be interesting to confirm the specificity of the BacT/Alert method using different gauges at diverse temperatures. However, most of the laboratories do not possess an automated blood culture system that allows simultaneous incubations at different temperatures. Also, the use of such systems requires higher amounts of samples to be tested and, as stated above, some ATMPs are produced in limited quantities, which means that not enough material may be available to perform several sterility tests at different temperatures.

In addition, but not less important, all of the operations performed by the operators using the BacT/Alert system guarantee the data incorruptibility and audit trails, as required by the Code of Federal Regulations (CFR) 21 part 11 regulations^31^ and by Annex 11 of the Volume 4 of GMP Medicinal Products for Human and Veterinary Use^32^ for GMP QC activities.

In the light of our results, we have confirmed that the BacT/Alert system can be used in GMP control activities to test the sterility of ATMPs, as shown previously.^4^^,^^10^^,^^14^ We demonstrated that the use of 27G needles decreases the TTD of most of the tested microorganisms, when compared to the Eur Ph method. Also, the use of 27G needles allows less air intrusion and, consequently, improves the growth and sensitivity of the method for anaerobic microorganisms, and we plan to validate this strategy using our in-house ATMPs in the future. We think that our work is of interest for researchers and professionals who must confirm the quality and safety of their biological products using automated blood culture systems.

## Materials and methods

### Microorganism suspension preparations, titer determination, and identification

American Type Culture Collection (ATCC) strains (Table 1) were purchased from Microbiologics (St. Cloud, MN, USA) and supplied as quantitative lyophilized pellets. Stock suspensions of all microorganisms with a theoretical concentration of 10 ≤ CFU/mL <100 and subsequent four dilutions were prepared in peptone water (Becton Dickinson [BD], Franklin Lakes, NJ, USA). Before and during the validation phases, viability, purity, and titer determination were assessed by plating 100 μL of each diluted suspension in triplicate on TSA (for aerobic suspensions), Columbia agar with 5% sheep blood (for anaerobic suspensions), and Sabouraud dextrose agar (SDA; for fungal suspensions) plates (all from BD). Plates were incubated at 32.5°C ± 2.5°C for bacteria and 22.5°C ± 2.5°C for fungi. Before incubation, agar blood plates with the anaerobic microorganisms were placed inside resealable GasPak EZ anaerobe pouch systems (BD). The CFU were quantified after 2–3 days for bacteria and 4–5 days for yeast and mold. For each experiment, the theoretical titer of each diluted suspension was confirmed by calculating the arithmetic average of the CFU values obtained. Purity and identity were determined as described elsewhere.^33^

### Validation strategy

To validate the method based on the BacT/Alert system (Biomérieux), we performed this system in parallel with the compendial direct inoculation sterility Eur Ph method. We used nine microorganisms detailed in Eur Ph 2.6.27^5^ and *S. epidermidis*, a potential environmental contaminant (Table 1). For sample preparation, 5-mL sterile conical-bottom tubes (Eppendorf, Hamburg, Germany) were used. For sample inoculation, 21G (BD) and 27G (B. Braun, Melsungen, Germany) needles were tested to assess their potential effect on microorganism growth. For each microorganism, the results obtained with the BacT/Alert method and the golden Eur Ph standard method were compared to evaluate the effectiveness and comparability of the former.

### Compendial direct inoculation sterility method

The compendial Eur Ph method was done as described elsewhere.^5^^,^^21^ Briefly, we examined the growth properties of the microorganisms detailed in Table 1 in TSB (BD, catalog no. 299416, 100-mL bottle, septum/screw cap) for aerobic bacteria and fungi and in FTM (BD, catalog no. 299417, 100-mL bottle, septum/screw cap) for anaerobic bacteria. Eur Ph recommends an incubation temperature of 22.5°C for growth of aerobic microorganisms in TSB.^5^^,^^21^ However, we and others^14^ have observed that most of the reference microorganisms used grow faster when incubated at 32.5°C (Table S8), leading us to choose this temperature for incubation of microorganisms using the Eur Ph method.

**Figure 1:**
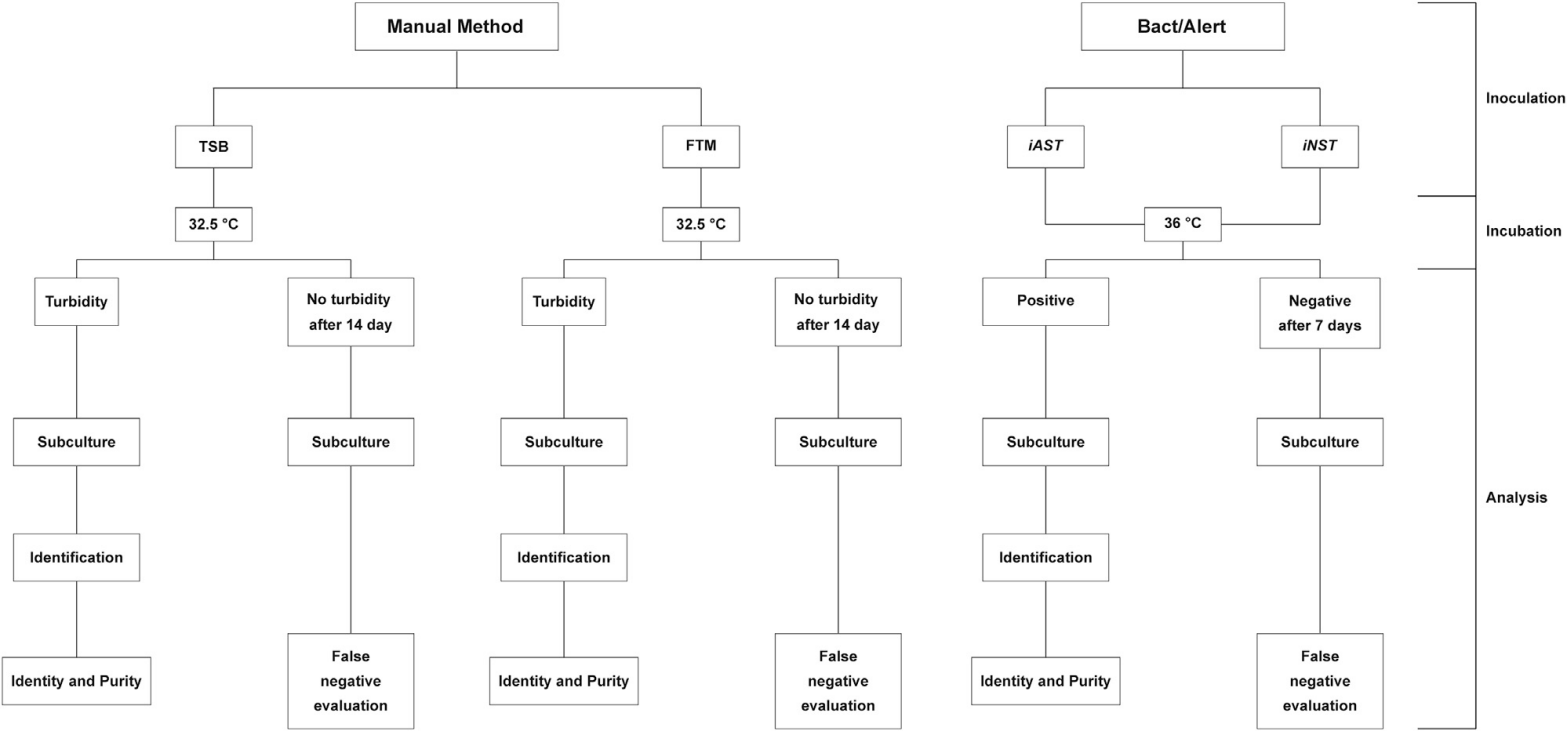
Scheme of the validation protocol Reference microorganisms were inoculated into each bottle using 21G or 27G needle, 2011Reference microorganisms were inoculated into each bottle using 21G or 27G needle. Manual method samples were incubated until positive or up to 14 days. BacT/Alert bottles were incubated until positive or up to 7 days.

Medium growth ability was evaluated by assessing its turbidity every 24 h for 14 days. When turbidity was observed, the liquid media were subcultured in the corresponding solid media for 2–5 days to evaluate the identity and purity of the corresponding microorganism. After 14 days, if turbidity was not observed, the sample was subcultured in solid culture media for 2–5 days to ensure that it was not a false negative (Figure 1). When microbial growth was observed in solid media but not previously in liquid media, we considered such a result a false negative. The microorganism grown in the plate was further identified by MALDI-TOF to confirm if it was the strain inoculated initially or a potential contaminant. If the former is verified, the cellular matrix would be evaluated for its antimicrobial properties. Regardless, to ensure the sterility of an ATMP and, consequently, the safety of the patient, the tests would be repeated and, if necessary, some incubation parameters optimized, such as temperature or period.

### Alternative BacT/Alert-based method

Among the different media available, we chose to use *iAST* and *iNST* bottles (both from Biomérieux), which do not contain antibiotics. These media were selected since the presence of antibiotics in ATMPs is undesirable,^2^^,^^28^ and it could affect the growth of the selected microorganisms during performance of the alternative method. Briefly, we examined the growth properties of the microorganisms detailed in Table 1 in *iAST* (aerobic bacteria and fungi) and *iNST* (anaerobic bacteria) bottles. Growth of microorganisms was performed at 36°C, as shown by BacT/Alert readings and an external calibrated GMP thermometer, in agreement with the temperature range (35°C–37°C) recommended by the Eur Ph.^6^ Growth properties were assessed automatically by BacT/Alert software, which reported the TTD, i.e., the time at which the instrument detected microorganism growth for the first time. Every 24 h, if positivity was detected, an aliquot of the sample was subcultured in solid media to evaluate the identity and purity of the inoculated suspension. After 7 days, negative samples were subcultured in solid media for 2–5 days to evaluate potential false negative detection, as described above for the compendial Eur Ph method.

### Validation parameters

Since these methods are qualitative tests, we wanted to demonstrate the following parameters: specificity, detection limit, ruggedness, repeatability, and cross-reaction contamination.^6^^,^^8^^,^^34^

#### Specificity

The specificity of an alternative qualitative microbiological method is the ability to detect the specific presence of one or more microorganisms present in the test. To verify this method capability, each operator tested the batch of each media inoculating in triplicate one microorganism at a time, with a concentration of 25–50 CFU, and tested the several microbiological media for growth ability.^5^^,^^21^

#### Detection limit

The lowest number of microorganisms that an analytical method can detect is defined as the detection limit. In this study, we chose a detection limit of 50% of total inoculated samples, as suggested elsewhere^6^, ^7^, ^8^^,^^21^^,^^22^^,^^25^ (i.e., the minimum number of samples where at least 50% of the growth of microorganisms was observed). To determine the detection limit, we performed four independent analyses, testing three repetitions of microbial suspensions of 25–50, 5–10, 2–5, 1–2, and 0–1 CFU. The number of actual CFU inoculated in the bottles was confirmed by plating 100 μL of each dilution in the respective media agar plates

#### Ruggedness and repeatability

The ruggedness of a method is defined as the ability to provide reproducible results even when minimal expected variations are induced, such as performing the procedure on different days, use of different reagents, or execution by different operators. To evaluate the ruggedness of the BacT/Alert method, the experiments described above were conducted on 4 different days by four different operators, each using a different batch of microbiological culture medium. Repeatability is defined as the closeness or concordance between the results obtained from measurements made under the same experimental conditions. To demonstrate this parameter, each operator performed three different inoculations for each dilution of the microbial suspension on the same day.

#### Technical cross-contaminations

Technical cross-contaminations occur when samples are contaminated during preparation and/or inoculation. To ensure that the BacT/Alert alternative method was not prone to cross-reaction contaminations, for each dilution used we added one negative control, which was processed together with three positive samples. For each operator and for each microorganism, the evaluation involved the simultaneous handling and processing of 15 positive and 5 negative samples.

### Analytical method equivalency

There are two concepts to compare different analytical methods with the same goal: analytical method comparability and analytical method equivalency. Chambers et al.^35^ considered that analytical method comparability refers to studies that evaluate similarities and differences in method performance characteristics between two analytical methods (i.e., accuracy, precision, specificity, detection limit, and quantization limit), while analytical method equivalency is included in the analytical method comparability and evaluates similarities between two analytical methods, regarding their obtained results for the same sample. In other words, analytical method equivalency evaluates whether the new method can generate equivalent results to those obtained with an existing method. In a recent publication, Chatfield et al.^36^ suggested another way to differentiate these two concepts, where analytical method equivalency is restricted to a formal statistical analysis to evaluate similarities in method performance characteristics.

To assess the equivalence of the two methods, we ran them in parallel and determined the degree to which the alternative method showed equivalence to the pharmacopoeial method. In particular, we compared the rate of positive and negative results produced by the BacT/Alert method versus the Eur Ph method for identical samples.^6^

### Data analysis

To assess whether the BacT/Alert system was at least equivalent to the golden Eur Ph standard method regarding the growth of microorganisms at low-level inoculation (25–50 CFU), we used the χ^2^ test. We compared the number of positive cultures detected with each method using the same gauge type. Normality of data and homogeneity of variances were tested with a graphical approach, using a Q-Q plot and F test statistics, respectively. The t test was used to compare the TTD values needed to detect microbial growth with the two methods, both (1) for the results obtained for each medium using the two different gauges, and (2) the different media using 21G or 27G needles. STATA 15.1 statistical software (StataCorp, College Station, TX, USA) was used for all statistical analysis. We considered a value of p <0.05 statistically significant.

## Supplemental information

Document S1. Tables S1–S10 and Supplemental materials and methods

Document S2. Article plus supplemental information
