# Supplementary material for: Use of 27G needles improves sensitivity and performance of ATCC anaerobe reference microorganism detection in BacT/Alert system
Source: Mol Ther Methods Clin Dev. 2021 Jan 26;20:542–50. doi: 10.1016/j.omtm.2021.01.010 (PMC7890369; doi:10.1016/j.omtm.2021.01.010)
Supplement: Document S1. Tables S1–S10 and Supplemental materials and methods [file mmc1.pdf]

OMTM, Volume *20*

Supplemental Information

Use of 27G needles improves sensitivity and performance of ATCC anaerobe reference microorganism detection in BacT/Alert system

Salvatore Pasqua, Giampiero Vitale, Anna Pasquariello, Bruno Douradinha, Fabio Tuzzolino, Francesca Cardinale, Chiara Cusimano, Chiara Di Bartolo, Pier Giulio Conaldi, and Danilo D'Apolito

Supplemental Tables

Table S1 – Recovery and TTD (Time To Detection) for *Propionibacterium acnes*.

Range of total CFU inoculated		25-50			
**Microorganism**	**Colony Counts (CFU)**	**Gauge**	**Recovery**	**TTD (h) ± SD[a]**	***p*[b]**
***Propionibacterium acnes* (*iNST*)**	39	**21**	12/12	88.8 ± 11.59	**0.0014**
		**27**	12/12	74 ± 6.92	
Propionibacterium acnes (FTM)		**21**	12/12	196 ± 9.79	0.5575
		**27**	12/12	200 ± 12.39	

[a] SD, Standard Deviation

[b] Statistically significant values are in bold

Table S2 – LOD (Limit of Detection) for microorganisms in aerobic media

Range of total CFU inoculated		25-50	5-10	2-5	1-2	0-1	LOD observed[a]
**Microorganism**	**Gauge**	**Recovery**	**Recovery**	**Recovery**	**Recovery**	**Recovery**	
***S. aureus* (*iAST*)**	**21**	12/12	12/12	9/12	6/12	2/12	**1-2**
	**27**	12/12	12/12	12/12	8/12	3/12	**1-2**
S. aureus (TSB)	**21**	12/12	12/12	10/12	10/12	5/12	**1-2**
	**27**	12/12	12/12	12/12	6/12	3/12	**1-2**
P. aeruginosa (iAST)	**21**	12/12	12/12	9/12	6/12	0/12	**1-2**
	**27**	12/12	12/12	12/12	6/12	0/12	**1-2**
P. aeruginosa (TSB)	**21**	12/12	12/12	6/12	4/12	0/12	**2-5**
	**27**	12/12	12/12	7/12	5/12	1/12	**2-5**
B. subtilis (iAST)	**21**	12/12	12/12	12/12	10/12	5/12	**1-2**
	**27**	12/12	12/12	12/12	10/12	5/12	**1-2**
B. subtilis(TSB)	**21**	12/12	12/12	11/12	9/12	2/12	**1-2**
	**27**	12/12	12/12	11/12	8/12	2/12	**1-2**
S. epidermidis (iAST)	**21**	12/12	12/12	12/12	1/12	1/12	**2-5**
	**27**	12/12	12/12	12/12	2/12	3/12	**2-5**
S. epidermidis (TSB)	**21**	12/12	12/12	9/12	0/12	0/12	**2-5**
	**27**	12/12	12/12	9/12	1/12	0/12	**2-5**
S. pyogenes (iAST)	**21**	12/12	12/12	7/12	4/12	0/12	**2-5**
	**27**	12/12	12/12	9/12	6/12	2/12	**1-2**
S. pyogenes (TSB)	**21**	12/12	12/12	12/12	6/12	0/12	**1-2**
	**27**	12/12	12/12	11/12	11/12	1/12	**1-2**
C. albicans (iAST)	**21**	12/12	12/12	12/12	12/12	0/12	**1-2**
	**27**	12/12	12/12	12/12	12/12	2/12	**1-2**
C. albicans (TSB)	**21**	12/12	12/12	12/12	12/12	0/12	**1-2**
	**27**	12/12	12/12	12/12	12/12	0/12	**1-2**
A. brasiliensis (iAST)	**21**	12/12	12/12	12/12	8/12	0/12	**1-2**
	**27**	12/12	12/12	12/12	8/12	0/12	**1-2**
A. brasiliensis (TSB)	**21**	12/12	12/12	12/12	12/12	0/12	**1-2**
	**27**	12/12	12/12	12/12	10/12	0/12	**1-2**

[a] LOD observed values are in bold

Table S3 – LOD (Limit of Detection) for microorganisms in anaerobic media

Range of total CFU inoculated		25-50	5-10	2-5	1-2	0-1	LOD observed[a]
**Microorganism**	**Gauge**	**Recovery**	**Recovery**	**Recovery**	**Recovery**	**Recovery**	
B. fragilis (iNST)	**21**	5/12	3/12	3/12	0/12	0/12	----
	**27**	12/12	12/12	8/12	5/12	0/12	**2-5**
B. fragilis (FTM)	**21**	12/12	12/12	10/12	5/12	4/12	**2-5**
	**27**	12/12	8/12	7/12	4/12	0/12	**2-5**
C. sporogenes (iNST)	**21**	12/12	12/12	11/12	5/12	0/12	**2-5**
	**27**	12/12	12/12	12/12	12/12	2/12	**1-2**
C. sporogenes (FTM)	**21**	12/12	12/12	12/12	12/12	0/12	**1-2**
	**27**	12/12	12/12	12/12	12/12	4/12	**1-2**
S. pyogenes (iNST)	**21**	12/12	12/12	7/12	5/12	0/12	**2-5**
	**27**	12/12	12/12	12/12	8/12	3/12	**1-2**
S. pyogenes (FTM)	**21**	12/12	12/12	12/12	6/12	0/12	**1-2**
	**27**	12/12	12/12	12/12	7/12	1/12	**1-2**

[a] LOD observed values are in bold

Table S4 – Types of containers evaluated for our validation protocol

Candidate	Supplier	Height	Width
**5 ml sterile conical bottom tube**	Eppendorf, Hamburg, Germany	5.5 cm	1.5 cm
**15 ml sterile conical bottom tube**	Corning, New York, USA	11.7 cm	1.7 cm
**50 ml sterile conical bottom tube**	Corning, New York, USA	11.5 cm	2.8 cm
**50 ml sterile flat bottom**	Syntesys S.a.s., Teolo, Italy	6.9 cm	5.5 cm

Table S5 – Needles used in our validation protocol.

Needle	Supplier	Height	Width
**Microlance 21 gauge**	BD	4 cm	0.8 cm
**Sterican 23 gauge**	B.BRAUN (Melsungen, Germany)	6 cm	0.6 cm
**Sterican 25 gauge**		4 cm	0.5 cm
**Sterican 27 gauge**		4 cm	0.4 cm

Table S6 – GPT for *B. subtilis* in TSB using 50 ml conical bottom tubes

Microorganism	Colony Counts (CFU)	Gauge	Recovery[a]	TTD (h) ± SD[b]
***B. subtilis* (25-50)**	40	21	9/9	48 ± 0
***B. subtilis* (5-10)**	8		9/9	48 ± 0
***B. subtilis* (2-5)**	3		6/9	48 ± 0

[a] Recovery was defined as the ratio of number of bottles which display microbial growth by the total number of inoculated bottles

[b] SD, Standard Deviation

Table S7 – GPT for *B. subtilis* in TSB using 5 ml conical bottom tubes and 50 ml flat bottom containers

Microorganism	Tube	Colony Counts (CFU)	Gauge	Recovery	TTD (h) ± SD[a]
***B. subtilis* (25-50)**	5 ml conical	39	21	9/9	48 ± 0
	50 ml flat bottom			9/9	48 ± 0
***B. subtilis* (5-10)**	5 ml conical	7		9/9	48 ± 0
	50 ml flat bottom			7/9	48 ± 0
***B. subtilis* (2-5)**	5 ml conical	4		7/9	48 ± 0
	50 ml flat bottom			4/9	48 ± 0

[a] SD, Standard Deviation

Table S8 – Statistical analysis of TTD (Time To Detection) for microorganisms in TSB incubated at 32.5 ± 2.5 °C or at 22.5 ± 2.5 °C.

Compendial Method						
**Microorganism**	**Organism Type**	**Colony Counts (CFU)**	**Gauge**	**32.5 ± 2.5°C**	**22.5 ± 2.5°C**	***p***
				**TTD (h) ± SD[a]**	**TTD (h) ± SD[a]**	
Staphylococcus aureus	Gram-Positive	48	21	32 ± 11.3	48 ± 0	**0.0001**
Pseudomonas aeruginosa	Gram-Negative	42		48 ± 0	58 ± 15.36	**0.0420**
Bacillus subtilis	Gram-Positive	39		48 ± 0	52 ± 8.94	0.1521
Staphylococcus epidermidis	Gram-Positive	41		144 ± 0	152 ± 19.37	0.1521
S. pyogenes	Gram-Positive	41		48 ± 0	72 ± 0	**0.0000**
Candida albicans	Yeast	43		90 ± 10.39	98 ± 6.63	**0.0426**
Aspergillus brasiliensis	Mold	39		88 ± 11.31	100 ± 8.94	**0.0114**

[a] SD, Standard Deviation

[b] Statistically significant values are in bold

Table S9 – Recovery and TTD (Time To Detection) for microorganisms in anaerobic media inoculated with 21, 23, 25, 27 gauge needles

**Range of total CFU inoculated**		**25-50**	
**Microorganism**	**Gauge**	**Recovery**	**TTD (h) ± SD[a]**
B. fragilis (iNST)	**21**	2/9	96 ± 0
	**23**	4/9	84 ± 13.85
	**25**	6/9	72 ± 21.46
	**27**	9/9	64 ± 12
B. fragilis (FTM)	**21**	9/9	58.66 ± 12.65
	**23**	9/9	56 ± 12
	**25**	9/9	56 ± 16.97
	**27**	9/9	58 ± 16.1
C. sporogenes (iNST)	**21**	9/9	29.33 ± 10.58
	**23**	9/9	29.33 ± 10.58
	**25**	9/9	26.66 ± 8
	**27**	9/9	26.66 ± 8
C. sporogenes (FTM)	**21**	9/9	24 ± 0
	**23**	9/9	24 ± 0
	**25**	9/9	24 ± 0
	**27**	9/9	24 ± 0

[a] SD, Standard Deviation

Table S10 – Statistical analysis of TTD (Time To Detection) for microorganisms in BacT/Alert bottles seeded with 25-50 CFU and suspended in Peptone Water or Exhausted Culture Media.

		iAST		
**Microorganism**	**Colony Counts (CFU)**	**Peptone Water**	**Exhausted Culture media**	**Comparison of matrix (*p*)**
		**TTD (h) ± SD[a]**	**TTD (h) ± SD[a]**	
S. aureus	47	33.2 ± 2.1	33.7 ± 2.3	0.7210
P. aeruginosa	40	26.8 ± 1.8	27 ± 2	0.8264
S. epidermidis	39	30.2 ± 2.4	29.9 ± 2	0.8096
C. albicans	45	39.2 ± 1.6	40.2 ± 2.2	0.7894

		iNST		
**Microorganism**	**Colony Counts (CFU)**	**Peptone Water**	**Exhausted Culture media**	**Comparison of matrix (*p*)**
		**TTD (h) ± SD[a]**	**TTD (h) ± SD[a]**	
C. sporogenes	38	31.7 ± 2	30.8 ± 2	0.4643

[a] SD, Standard Deviation

Supplemental Methods

Pre-validation setup

Installation Qualification and Operational Qualification

As required by Good Manufacturing Practices (GMP) guidelines, [1,2] we performed the Installation Qualification (IQ) and the Operational Qualification (OQ). The former refers to a well documented verification procedure that all equipments have been correctly delivered, installed and configured in agreement with their supplier's instructions. Afterwards, an OQ must be done, to confirm that the equipment performance meets the user requirement specifications within the manufacturer-specific operating ranges.

Quality control of materials and reagents

Upon arrival, all materials and reagents were checked to confirm they meet the user required specifications. Microbiological media lots were evaluated for sterility and growth promotion properties. [3,4] Microbial reference strains (Table 1 of main manuscript) were verified and validated for identity, viability and titer, which were in agreement with their certificates of analyses (CoA) values. All quality control (QC) personnel were trained in accordance to the written analytical method validation protocol and standard operative procedures (SOP), as requested by GMP guidelines.

Containers, syringe and needles

The shape of the container in which bacterial suspensions are prepared was a critical factor for subsequent withdrawal of the referred suspensions. Thus, we performed tests with different types of containers (Table S4) to understand if they met the requirements of our validation.
In this work, we used Emerald syringes (BD; Franklin Lakes, NJ, USA) which, without needle, are 8.5 cm long and 1.8 cm wide. All the needles used in this work had a minimum height of 4.0 cm (Table S5).
The choice of the containers was done using the 21 gauge needles. The 15 ml conical bottom tubes were discarded since they do not allow full withdrawal of the suspension. On the other hand, 50 ml tubes with conical bottom do not allow proper introduction of bacterial suspensions into the tube itself, due to its height, and sterile withdrawal of bacterial suspensions from these tubes was not always possible. Preliminary Growth Promotion Tests (GPTs) were performed using these tubes, to prepare and inoculate *B. subtilis* in TSB bottles (Table S6), using different CFU ranges (2-5, 5-10 and 25-50). The microbial suspensions were prepared using 3 ml of peptone water (BD).
Overall, we observed growth of this bacterium in 24 out of 27 bottles. In 4 samples out of those 24 (1 of 2-5, 2 of 5-10 and 1 of 25-50 CFU), the simultaneous presence of another microorganism was observed. The subcultures of these positive bottles showed the presence of *B. subtilis* and a contaminant, *Staphylococcus epidermidis*, usually found in the skin. All procedural controls complied with what was expected, i.e., for each dilution used (i) no foreign microorganisms were found in solid media, (ii) the respective negative controls showed total absence of microbial growth and (iii) the subcultures of the 3 bottles in which no growth of *B. subtilis* was observed were also negative. Due to these results, we decided not to use this type of tubes in the validation. To identify a suitable type of container, we repeated the GPT with *B. subtilis* using 5 ml conical bottom and 50 ml flat bottom tubes (Table S7).
From an operational point of view, there were no difficulties since both type of tubes' length allowed the microbial suspension to be dispensed perfectly into the bottom of the container and an easy withdrawal of the microbial suspension mixture without touching the inner walls of the tube with the syringe. Using 5 ml conical tubes, the bottles inoculated with the suspension mixture with 5-10 and 25-50 CFU showed full recovery, while those inoculated with 2-5 CFU displayed a recovery of 77.77% (7/9). When flat bottom containers were utilized, we observed that inoculated bottles only showed 100 % of recovery for the 25-50 CFU suspension. The other 2 dilutions showed lower recovery, in particular 77.77% (7/9) and 44.44% (4/9) for 5-10 and 2-5 CFU, respectively. No cross-contamination was observed for both tubes and all dilutions used.
Considering that the procedures were performed in parallel with the same preparation of the microbial suspensions and with the same materials, this discrepancy in the recovery results was probably due to the dispersion of microorganisms in the flat bottom container when compared to the conical one. Since the latter concentrates the microbial suspension in a single point in the bottom, the collection of the suspension is maximized. For this reason we have chosen 5 ml conical bottom tubes for the validation procedure.

Incubation temperature for microbial growth in TSB using compendial Eur Ph method

An incubation temperature of 22.5 °C for growth of aerobic microorganisms in TSB is advised by the Eur Ph. [3] However, based in our previous experience and in another work, [5] a growth temperature of 32.5 °C allows faster growth of most of the reference aerobic microorganisms. Therefore, we performed a comparative analysis of the aerobic microorganisms growth in TSB incubated at either 22.5 °C or 32.5 °C. For each microorganism, we did 12 replicates. As expected, we observed that most of the reference microorganisms used grew significantly faster when incubated at

32.5°C (Table S8), prompting us to choose this temperature for microorganisms' incubation using the Eur Ph compendial method.

Needle gauge

Before beginning our validation protocol, and as suggested by, [3,4] we performed the GPTs for all reference microorganisms (Table 1 from main manuscript; 10-100 CFU) in their respective media, to confirm if the latter were suitable for microbial growth. Using TSB, FTM and *iAST* media, we observed full growth of the inoculated microorganisms confirming the media was adequate for the validation assay, as patent in the Tables 2 and 3 in the main manuscript. *C. sporogenes* and *S. pyogenes* displayed full growth in *iNST*, confirming its suitability for validation assays of these 2 anaerobic microorganisms' growth, as shown in Table 3 in the main manuscript. However, *B. fragilis* did not fully grow in *iNST* and, in these conditions, a validation cannot be performed with this medium. [4] These results were the same for all tested media lots. The initial GPTs were done using a 21 gauge needle and, as mentioned elsewhere, [6] the use of needles with larger diameters may introduce unwanted air into culture bottles, affecting negatively the growth of anaerobic bacteria, such as *B. fragilis*. Since the *iNST* medium supplier declared this medium was able to sustain the growth of *B. fragilis*, we decided to perform additional tests using different gauge needles for introduction of the microbial suspensions into *iNST* BacT/Alert bottles. The gauges used are described in Table S5. Usually, in routine diagnostics procedures, blood collection (venipuncture) and subsequent inoculation into bottles are done using needles with gauges between 19 and 25. However, for recovery of anaerobic microorganisms using BacT/Alert the suppliers suggest the use of 27 gauge needles for inoculation into iNST bottles. Thus. we have chosen to perform the validation by inoculating with both 21 (BD) and 23, 25 and 27 (all from B. Braun, Melsungen, Germany) gauge needles in parallel. We then performed the GPTs, inoculating the microorganisms with 21, 23, 25 or 27 gauge, on 9 iNST bottles and 9 FTM bottles. *C. sporogenes* was used as anaerobic microorganisms control. As shown in Table S9, only the 27 gauge allowed *B. fragilis* growth in all *iNST* bottles, as required by Eur Ph. [4] *C. sporogenes* grew in all iNST bottles, as expected. Furthermore, for *B. fragilis* grown in *iNST* medium, we observed a gradual decrease in Time to Detection (TTD), as the gauge decreases, suggesting that the anaerobic environment was less perturbed, as described elsewhere. [6] Remarkably, FTM was not affected by gauge size. After this evaluation, we chose to validate the alternative BacT/Alert method using 21 and 27 gauge needles.

Matrix choice

As suggested by GMP guidelines, [7] ATMPs should not contain antibiotics or antimicrobials, since their presence is undesired in the final product and to avoid interference during the sterility testing. In agreement, our ATMPs are produced without antibiotics or antimicrobials. [8] Thus, the matrix of choice for this validation should also be antibiotic free. Previous works have shown that the matrix itself does not affect microbial growth. [9,10] Prior to our validation procedures, we decided to compare 2 different matrices, exhausted culture media derived from our ATMPs production [8] and peptone water. Thus, we assessed the recovery and the TTD of several microorganisms using these 2 matrices. As before, we did 12 replicates for each microorganism. The recovery and the TTD obtained can be observed in Table S10. As expected, no significant difference was observed for these parameters using either exhausted culture media or peptone water. Our results are in accordance with previous works, in which validations were performed with either complex matrices such as cellular products or simple matrices, like saline solution, with no significant differences among them. [9,10] Thus, we chose peptone water for all microbial suspensions used during the validation procedures.

Supplemental References

1. European Commission (2015). EudraLex - Volume 4 - Good Manufacturing Practice (GMP) guidelines. https://ec.europa.eu/health/documents/eudralex/vol-4_en. Accessed 23/10/2020.
2. European Commission (2008). Annex 11 - Computerized Systems. EudraLex Guidelines for good manufacturing practices for medicinal products for human and veterinary use.
3. European Commission (2017). 2.6.1 Sterility. In European Pharmacopoeia 9.2th Edition (European Union).
4. Council of Europe (2017). 2.6.27 Microbial Examination of cell-based Preparations. In European Pharmacopoeia 9.2th Edition (European Union).
5. England, M.R., Stock, F., Gebo, J.E.T., Frank, K.M., and Lau, A.F. (2019). Comprehensive Evaluation of Compendial USP<71>, BacT/Alert Dual-T, and Bactec FX for Detection of Product Sterility Testing Contaminants. J Clin Microbiol *57*.
6. Biomerieux (2014). BACT/ALERT® i NST. https://techlib.biomerieux.com/wcm/techlib/techlib/documents/docLink/Package_Insert/81642001-81643000/Package_Insert_-_9308859_-_B_-_en_-_259785.pdf. Accessed 14/12/2020.
7. Commission, E. (2017). Guidelines on Good Manufacturing Practice specific to Advanced Therapy Medicinal Products.
8. D'Apolito, D., D'Aiello, L., Pasqua, S., Pecoraro, L., Barbera, F., Douradinha, B., Di Martino, G., Di Bartolo, C., and Conaldi, P.G. (2020). Strategy and validation of a consistent and reproducible nucleic acid technique for mycoplasma detection in advanced therapy medicinal products. Biologicals.
9. Khuu, H.M., Stock, F., McGann, M., Carter, C.S., Atkins, J.W., Murray, P.R., and Read, E.J. (2004). Comparison of automated culture systems with a CFR/USP-compliant method for sterility testing of cell-therapy products. Cytotherapy *6*, 183–195.
10. Hocquet, D., Sauget, M., Roussel, S., Malugani, C., Pouthier, F., Morel, P., Gbaguidi-Haore, H., Bertrand, X., and Grenouillet, F. (2014). Validation of an automated blood culture system for sterility testing of cell therapy products. Cytotherapy *16*, 692–698.
